# Automatic Removal of Physiological Artifacts in EEG: The Optimized Fingerprint Method for Sports Science Applications

**DOI:** 10.3389/fnhum.2018.00096

**Published:** 2018-03-21

**Authors:** David B. Stone, Gabriella Tamburro, Patrique Fiedler, Jens Haueisen, Silvia Comani

**Affiliations:** ^1^Department of Neuroscience, Imaging and Clinical Sciences, Behavioral Imaging and Neural Dynamics Center, Università degli Studi G. d'Annunzio Chieti e Pescara, Chieti, Italy; ^2^Institute of Biomedical Engineering and Informatics, Technische Universität Ilmenau, Ilmenau, Germany

**Keywords:** EEG, artifact removal, ICA, support vector machine, eyeblink artifact, eye movement artifact, myogenic artifact, sports science

## Abstract

Data contamination due to physiological artifacts such as those generated by eyeblinks, eye movements, and muscle activity continues to be a central concern in the acquisition and analysis of electroencephalographic (EEG) data. This issue is further compounded in EEG sports science applications where the presence of artifacts is notoriously difficult to control because behaviors that generate these interferences are often the behaviors under investigation. Therefore, there is a need to develop effective and efficient methods to identify physiological artifacts in EEG recordings during sports applications so that they can be isolated from cerebral activity related to the activities of interest. We have developed an EEG artifact detection model, the Fingerprint Method, which identifies different spatial, temporal, spectral, and statistical features indicative of physiological artifacts and uses these features to automatically classify artifactual independent components in EEG based on a machine leaning approach. Here, we optimized our method using artifact-rich training data and a procedure to determine which features were best suited to identify eyeblinks, eye movements, and muscle artifacts. We then applied our model to an experimental dataset collected during endurance cycling. Results reveal that unique sets of features are suitable for the detection of distinct types of artifacts and that the Optimized Fingerprint Method was able to correctly identify over 90% of the artifactual components with physiological origin present in the experimental data. These results represent a significant advancement in the search for effective means to address artifact contamination in EEG sports science applications.

## Introduction

A continuing challenge in the acquisition and analysis of human electroencephalographic data (EEG) is the presence of artifacts–electrical activity generated outside of cerebral sources of primary interest. Artifacts can arise from a number of sources including those of extracerebral physiological origin, such as those generated from eyeblinks, eye movements, and myogenic activity due to muscle activity in the head and neck, as well as non-physiological sources, such as electrical interference from external power sources and mechanical artifacts generated from electrode displacement and EEG equipment disturbances.

A number of methods have been proposed to address the presence of artifacts, including methods that attempt to minimize the artifactual sources during EEG acquisitions, as well as a variety of methods that try to reduce or remove artifacts from EEG data after acquisition and prior to further processing and analysis, including blind source artifact separation methods, co-acquisition of electromyogenic and ocular activity for post-acquisition removal, visual inspection of EEG data and manual deletion of artifactual data segments (for recent reviews see Urigüen and Garcia-Zapirain, 2015; Islam et al., 2016). Many of these methods rely on the participants who are asked to limit the number of artifacts they generate or on the investigator who inspects the data and relies on his or her experience to identify artifacts and remove them. As a result, in recent years efforts have been made to develop algorithms to automatically detect and remove artifacts in EEG data (e.g., Barbati et al., 2004; Viola et al., 2009; Mognon et al., 2011; Zou et al., 2016; Radüntz et al., 2017). Although these methods have proven effective in certain applications and under well-defined conditions, no universal method is agreed upon and none has proven completely effective.

We recently developed a system for the automatic classification and removal of the most ubiquitous physiological artifacts–eyeblinks, eye movements, and muscle and cardiac artifacts (Tamburro et al., 2018). Our method, the Fingerprint Method, was developed from both existing methods and novel approaches. The underlying principle of the Fingerprint Method was to use a variety of features of the independent components separated from the EEG data, that span spatial, temporal, spectral, and statistical domains to classify the components of artifactual origin. The independent components containing the artifacts are then disregarded in the reconstruction of the artifact-free EEG. For the automatic classification of artifactual components we used a common machine learning algorithm where these sets of features, the “fingerprints,” were used to build a model of each artifact type. Our initial findings proved that our method was successful in discriminating artifactual and non-artifactual components and achieved accuracies comparable or superior to those of other automatic artifact classification methods. In addition, we demonstrated that the Fingerprint Method was able to successfully identify artifacts in EEG data acquired with different EEG acquisition systems, and variable electrode types, numbers, and layouts. Further, the Fingerprint Method was able to detect artifactual components even when the total number of separated independent components was changed: artifactual components were successfully identified whether EEG data were decomposed into a small number (20) or a larger number (50 or 80) of independent components.

The primary purpose of the current study was to optimize the Fingerprint Method by refining the set of fingerprint features designed to identify each type of artifact. The original Fingerprint Method employed a set of 14 features to classify artifactual components regardless of the type of artifact investigated. Some of these features were designed to detect a specific type of artifact (such as eyeblinks or cardiac interference), while others were designed to capture more general characteristics of all artifact types. In the present study we sought to develop a set of unique classifiers optimally tuned to detect each specific artifact by employing a method to (1) critically determine which particular features were best suited to detect each artifact type based on the classifier's performance, and (2) to remove those features which performed ineffectively or even detrimentally. Thus, the Optimized Fingerprint Method retains only those features which are proven to best discriminate each type of physiological artifact. Our motivation for optimizing the Fingerprint Method was based on several assumptions. First, it is a well-known postulate of statistics and machine learning that models with too many parameters suffer from “overfitting” and may not generalize well in classifying new data. Second, including features that have low discriminability between artifactual and non-artifactual data components adds noise to our model. Such features act as random variables that reduce the efficiency and efficacy of our algorithm. Third, a smaller set of features would improve computational efficiency. Since the quantification of each feature incurs a computational “cost,” fewer features would result in shorter processing times and a reduced allocation of computational resources, which could prove practically beneficial, particularly when applied to large datasets or prospective online applications. Finally, retaining a smaller number of features may make our model more tractable and comprehensible. Possessing a more intuitive understanding of the model facilitates further improvements and a more direct interpretation of the results.

To facilitate the optimization of the Fingerprint Method, we have increased the number of EEG datasets used to build and test our model. In extension to our previous findings, we have included data acquired from different EEG systems with different numbers, types and layouts of electrodes, and we have decomposed each dataset into the number of independent components which achieved the highest number of clear artifactual and non-artifactual components.

Once we determined the optimal features to include in our model, we tested it on experimental data derived from a sports science paradigm. EEG applications in sports science have become more common in recent years and have provided new insights into the nature and improvement of athlete performance. This effort has been aided by recent advances in EEG data acquisition, including advances in hardware portability and comfort, as well as reduced preparation times (Park et al., 2015). While these enhancements have made possible the practical application of EEG in sports science, acquiring informative EEG data from participants actively engaged in physical activity continues to present a number of challenges (Thompson et al., 2008). A primary source of difficulty is the presence of physiological artifacts. Although they can be detrimental in all EEG applications, limiting the occurrence of these artifacts and finding effective means for removing them are particularly challenging in sports applications for several reasons. It is difficult or impossible to ask participants to limit the number of muscle artifacts they generate given that muscle activity is inherent in the task being investigated. Depending on the sport activity under investigation, muscular contractions can be more intense, frequent, and chronic than those occurring in more traditional EEG settings. Myogenic artifacts are generated by contractions of the head, neck and facial muscles. Primary head and neck movements are often necessary in many sports activities (e.g., to track a moving target such as a tennis ball), but secondary movement of the head and neck also occur to stabilize the body during other movement activities (e.g., during walking or cycling; Gwin et al., 2011). Additionally, contractions of the facial muscles are difficult to avoid in many sports applications (e.g., the grimace of a weight-lifter; Reis et al., 2014). Eyeblinks and eye movements are also difficult to limit because of task requirements, sweating and participant attention fully focused on performance rather than the recording conditions. Moreover, eyeblink rate has been reported to be correlated with fatigue, which is inevitable in many sports applications (Stern et al., 1994). Artifacts of non-physiological origin can also interfere with the detection of physiological artifacts in sports applications. Electrode displacement, cable movement and tension artifacts, as well as signal drop-out due to excessive sweating and movement, can degrade signal quality rendering identification of physiological artifacts more difficult. Because of the differing electrolyte composition present in human sweat, extensive sweating can cause a low frequency artifact and alters electrode-skin impedance (Reis et al., 2014). Also, in sports involving a high degree of motion, gross motor movements can alter electrode-skin contact, resulting in sudden increase in electrode impedance, dramatic reduction of signal quality, and significant data loss (Thompson et al., 2008). Although the focus of the present study is on physiological artifacts, the presence of non-physiological disturbances can obscure not only brain signal but also the correct identification and removal of physiological noise sources. Thus, finding an effective means of identifying physiological artifacts and separating them from brain activity in sports science applications promises to considerably advance the field and EEG signal processing in general.

In what follows, we first present the optimization of our Fingerprint Method to automatically classify physiological artifacts in a set of cued artifactual testing data. The optimization procedure includes the selection of optimal features for the identification of each artifact type. Then, we demonstrate the Optimized Fingerprint Method in the detection of artifacts in experimental data acquired during sports performance. We have limited our scope to the automatic identification of three common physiological artifacts: eyeblinks, lateral eye movements, and myogenic artifacts generated from myogenic activity in facial, jaw, head and neck muscles.

## Materials and methods

### Human participants

Seventeen male non-clinical volunteers (age 28 ± 4 years) participated in the acquisition of the artifact-rich training and testing EEG data that were used to optimize the Fingerprint Method. Twenty-two male athletes (age 27 ± 7 years) volunteered in the cycling performance experiment that provided the EEG datasets that were used to validate the Optimized Fingerprint Method. Both acquisition studies were approved by the local Ethics Committee and complied with the ethical standards outlined in the Declaration of Helsinki. All volunteers gave written informed consent prior to participation. Participants had no known record of neurological, psychological, dermatological or ophthalmological diseases and were not under pharmacological treatment. Participants in the cycling performance experiment also provided medical certifications of fitness for participation to non-competitive sports activities and regularly practiced cycling at least twice a week.

### Development and testing of the optimized fingerprint method

The development of the Optimized Fingerprint Method was performed in several steps. First, artifact-rich EEG data were acquired in which participants generated multiple occurrences of each of the different artifact types of interest. Second, these data were decomposed into a set of independent components (ICs) by applying independent component analysis (ICA). Third, each IC was classified and labeled as being artifactual or non-artifactual by an expert investigator, and all labels were verified by an additional expert investigator. Next, for each IC (artifactual and non-artifactual), the values of the original set of 14 temporal, spatial, spectral, and statistical features were calculated. Based on these sets of features—which we refer to as “fingerprints”—and the labels provided, the ICs were sorted into artifactual and non-artifactual classes. We then trained a set of automatic classifiers, nonlinear binary support vector machines (SVMs) with a radial basis function kernel, to automatically identify each IC as artifactual or non-artifactual based on the values of its fingerprint features. Finally, for each artifact type we tested each classifier on artifact-rich EEG data using varying subsets of the fingerprint features, and determined the particular set of features which optimally identified artifactual and non-artifactual ICs. A final set of classifiers, one for each artifact type, was then constructed which classified based on these optimal sets of features.

#### Artifact EEG data acquisition

EEG data used to develop the Optimized Fingerprint Method were recorded in separate sessions using two different electrode systems. In the first system, a standard gel-based electrode cap consisting of 128 conventional Ag/AgCl electrodes arranged in a quasi-equidistant montage was used (Waveguard, Advanced Neuro Technologies B.V., Enschede, Netherlands; see Figure S1). Electrolytic gel was applied at each electrode location to ensure good electric contact with the scalp (ECI-Electrogel, Electrocap International Inc., Eaton, Ohio, USA). The second EEG system utilized a novel set of 97 dry multipin polyurethane electrodes with a Ag/AgCl coating arranged in an equidistant montage (Fiedler et al., 2015; see Figure S1). This system was developed to permit contact and conductivity between the scalp and the electrodes without the application of electrolytic gel or paste. For all acquisitions, signals from both EEG systems were recorded using a unipolar biosignal amplifier with common average reference (RefaExt, Advanced Neuro Technologies B.V., Enschede, Netherlands). All EEG datasets were acquired at 2048 Hz sampling frequency except for 16 datasets (8 gel-based, 8 dry electrode datasets) acquired at 1,024 Hz. All EEG datasets acquired at 2,048 Hz were downsampled at 1,024 Hz before further processing. A gelled Ag/AgCl electrode applied over the right mastoid served as ground.

EEG data were acquired in separates sessions for each of the three artifact types–eyeblinks, eye movements, and myogenic artifacts. During the eyeblink and eye movement artifact acquisition sessions, participants were seated at a distance of 50 cm in front of a 16:9 monitor with 30-inch diameter (Myrica V30–1, Fujitsu Siemens, Munich, Germany). A fixed chin rest was used to ensure a consistent screen-eye distance and minimize head movement. During eyeblink sessions, participants were instructed to fixate on a stationary red cross (approximately 2 × 2 cm) displayed on the monitor. A beep tone presented at 2 or 5 s intervals cued participants to blink and return to fixation. During eye movement sessions, the position of the red cross moved transiently in a repeated, deterministic sequence with an initial center position followed by a repeated sequence of left side of screen movement, right side of screen movement, bottom side of screen movement, and top side of screen movement, returning to the center fixation point after each movement. Each movement corresponded to a position change of approximately 16° within the visual field of the participant. Participants were instructed to follow the position of the red cross on the screen. All movements from center fixation occurred at 2 s intervals.

Myogenic artifact data were collected for five different muscle movements in separate sessions. Muscle movements included jaw flexions (masseter muscle), eyebrow and forehead flexions (procerus and frontalis muscle), as well as neck movements (splenius and suboccipital muscles) resulting in side-to-side head tilts and forward-backward head tilts. For all myogenic artifact acquisitions, participants were seated facing forward and instructed to initially relax the relevant muscles. For the acquisition of jaw, eyebrow, and forehead flexions, participants were instructed to contract the relevant muscle at maximal tension after an initial cue (amplitude of the myogenic interference is determined in the electrodes close to the relevant muscle) or, after some training, to maintain a contraction of approximately 80% of maximal tension. Cues were presented at 3 s intervals. Participants could relax the relevant muscle following a second cue delivered 3 s later. For neck movement acquisitions, participants were instructed to produce a series of movement sequences following an initiating cue. For side-to-side movements, participants began in an initial head forward position (center) followed by a left head tilt, center, right head tilt, center sequence. For forward-backward movements, participants began in an initial head forward position (center) followed by a chin down tilt, center, chin up tilt, center sequence. Movement initiating cues were presented every 3 s for all head movement sessions.

Cued artifact EEG data acquisitions occurred in two separate studies. During the first study, there were 20 acquisitions of eyeblink datasets (10 gel-based acquisitions, 10 dry electrode acquisitions) and 20 acquisitions of eye movement datasets (10 gel-based acquisitions, 10 dry electrode acquisitions). All 17 volunteers participated in the first study: seven volunteers provided eyeblink and eye movement data using only the gel-based system, seven volunteers provided eyeblink and eye movement data using only the dry electrode system, and three volunteers provided eyeblink and eye movement data using both the gel-based and dry electrode systems in separate sessions. In the second study, seven volunteers from the first study returned to provide two additional eyeblink datasets each (one gel-based acquisition, one dry electrode acquisition per volunteer) and two additional eye movement datasets each (one gel-based acquisition, one dry electrode acquisition per volunteer). Thus, there were a total of 34 (17 gel-based, 17 dry electrode) eyeblink and eye movement datasets acquired. The same seven volunteers also provided 10 muscular artifact datasets each (five gel-based acquisitions, one for each muscular artifact type, and five dry electrode acquisitions, one for each muscular artifact type) during the second study. Thus, there was a total of 70 (35 gel-based, 35 dry electrode) muscular artifact datasets acquired.

#### Data pre-processing and independent component analysis

EEG datasets acquired with a sampling frequency of 2,048 Hz were downsampled at 1,024 Hz. All EEG datasets were then filtered with a Butterworth bandpass filter with cut-off frequencies at 0.3 and 100 Hz. A notch filter at 50 Hz was applied to minimize noise from power line interference. EEG data were visually inspected, and EEG channels exhibiting isoelectric saturation, or poor scalp-surface contact, or excessive noise interference, were identified and excluded from further analysis (McMenamin et al., 2010). In cases where more than 20% of EEG channels exhibited excessive noise throughout the EEG time course, the entire dataset was excluded from further analysis. In cases where more than 50% of electrodes exhibited excessive noise during shorter time windows, those segments were trimmed from the data. Additionally, datasets where visual inspection revealed very few or no eyeblink, eye movement, or myogenic artifacts (depending on session type) were also excluded from further analysis.

Retained datasets were pre-whitened by Principal Components Analysis (PCA; Delorme et al., 2007) and decomposed into 20, 50, or 80 ICs using the extended Infomax ICA algorithm (Bell and Sejnowski, 1995; Lee et al., 1999). These decomposition levels were selected to mimic the most common clinical and experimental EEG conditions: 21 electrodes are typically used in a clinical setting, whereas commercial EEG caps for research purposes generally mount from 32 to 128 electrodes or more (Tamburro et al., 2018). For each EEG dataset, we retained for further analysis only the sets of separated ICs that contained clearly identifiable and non-redundant ICs. When applying ICA to cued eyeblink EEG datasets, for each dataset there were typically only one or two ICs containing eyeblinks, regardless of the total number of separated ICs (20, 50, or 80 ICs). This was due to the well-defined waveform of eyeblinks. Therefore, to increase the total number of artifactual eyeblink ICs available to train and test the SVM classifiers, for some eyeblink datasets we retained multiple sets of ICs decomposed at more than one level. For the eye movement and myogenic datasets, all levels of decomposition produced multiple artifactual ICs, so for each dataset we retained only one set of ICs (decomposed at either 20, 50, or 80 ICs; see the last row of Table 1 for the total number of artifactual ICs produced for each artifact type). Myogenic artifact datasets included all distinct types of triggered muscle artifacts: jaw tensions (10 datasets), eyebrow movements (5 datasets), forehead movements (4 datasets), and head tilts (9 datasets). All EEG data pre-processing and decomposition were performed using the EEGLAB toolbox (v. 13.6.5b; Delorme and Makeig, 2004). The main characteristics of the retained EEG datasets with cued artifacts used to develop the Optimized Fingerprint Method are summarized in Table 1.

**Table 1:** Artifact dataset characteristics.

	**Eye blink artifact datasets**	**Eye movement artifact datasets**	**Myogenic artifact datasets**
No. of Participants	17	17	7
No. of datasets acquired (gel-based/dry)	34 (17/17)	34 (17/17)	70 (35/35)
No. of datasets retained (gel-based/dry)	26 (15/11)	26 (16/10)	28 (23/5)
No. of ICs (20 IC/50 IC/80 IC)	2,030 (260/650/1120)	970 (260/550/160)	1,070 (260/650/160)
Ave. dataset length (SD)	239.33 (64.81)	117.55 (12.20)	65.56 (24.03)
No. of Artifactual ICs	49	214	325

*Average dataset length is given in seconds*.

#### Expert artifact classification and labeling

For each dataset containing a given artifact type (either eyeblink, or eye movement, or myogenic artifact), all ICs were labeled as either “artifact” or “non-artifact” by an experienced investigator who inspected the time course, topological plot, and power spectrum of each IC. All labels were independently verified by an additional investigator. For each dataset, labels identified only one artifact type (i.e., “eyeblink artifact,” “eye movement artifact,” or “myogenic artifact”). All other ICs, including ICs containing artifacts of a different type, were labeled as “non-artifact.”

#### Identification of fingerprint features

Fourteen different features were calculated for each IC from all datasets. A complete description of all features, including the calculation of each feature and the values of all parameters, is provided in Tamburro et al. (2018). We briefly describe the calculation of each feature below.

1. Temporal Kurtosis (K):

To calculate the Temporal Kurtosis Feature (K; Mognon et al., 2011) of each IC time course, the entire time courses of individual ICs were epoched into consecutive epochs of 5 s duration with a 1 s overlap. The mean amplitude of each IC time course epoch was then subtracted from each epoch. The Temporal Kurtosis, K was calculated according to Equation 1.

(1) $$\begin{array}{l} K=\frac{1}{m}\sum_{e=1}^{m}\left(\frac{\frac{1}{n}\sum_{i=1}^{n}{\left(s_{e,i}-\bar{s_{e}}\right)}^{4}}{{\left(\frac{1}{n}\sum_{i=1}^{n}{\left(s_{e,i}-\bar{s_{e}}\right)}^{2}\right)}^{2}}-3\right) \end{array}$$

where the parameters *s*_*i*_ denote the *i*th out of *n* data samples in the *e*th epoch data vector, and *m* is the number of epochs in the IC. Negative *K*-values were set to zero and all positive K values obtained were normalized to the maximum *K*-value across all ICs for each dataset.

2. Maximum Epoch Variance (MEV):

The Maximum Epoch Variance Feature (MEV; Mognon et al., 2011) of each IC time course was calculated according to Equation 2.

(2) $$\begin{array}{l} MEV=\frac{max{\left(\frac{1}{n}\sum_{i=1}^{n}{\left(s_{e,i}\right)}^{2}-{\left(\frac{1}{n}\sum_{i=1}^{n}s_{e,i}\right)}^{2}\right)}_{e}}{\frac{1}{m}\sum_{e=1}^{m}\left(\frac{1}{n}\sum_{i=1}^{n}{\left(s_{e,i}\right)}^{2}-{\left(\frac{1}{n}\sum_{i=1}^{n}s_{e,i}\right)}^{2}\right)} \end{array}$$

where the parameters *s*_*i*_, *i, n, e*, and *m* represent the same parameters as in Equation 1, and *max()*_*e*_ denotes the maximum across all epoch values. Epochs were defined in the same manner as described for the K feature. All MEV values obtained for all ICs were normalized with respect to the maximum MEV value across all ICs in a given dataset.

3. Spatial Average Difference (SAD):

The Spatial Average Difference Feature (SAD; Mognon et al., 2011) is based on disparities in the IC weights between frontal and posterior electrodes. SAD was calculated according to Equation 3.

(3) $$SAD=\;\left|\frac{1}{k}\sum_{e=1}^{k}a_{k,FA}\right|-\left|\frac{1}{k}\sum_{e=1}^{k}a_{k,PA}\right|$$

where *FA* refers to frontal area electrodes and includes the weights of electrodes whose angular positions range from 0 to 60°From the medial line and have a radial range ≥ 0.4, *PA* refers to posterior area electrodes and includes the weights of electrodes whose angular positions range from 0 to 120° From the medial line and have a radial range of 1.0, and *a* is the vector of the IC weights in the *k* electrode positions in *FA* and *PA*. For each IC, the variances of *FA* and *PA* electrode weights were calculated and the difference, variance(*FA*)–variance(*PA*), was determined. In cases where this difference was ≤ 0, it was assumed the SAD was due to a posterior source and was set to zero. Likewise, the average weights of left fronto-temporal electrodes and right fronto-temporal electrodes (regions defined below) were compared, and in cases where the averages were of opposite sign SAD was set to zero. SAD values were then normalized to the maximum SAD value obtained across all ICs for a given dataset.

4. The Spatial Eye Difference (SED):

The Spatial Eye Difference Feature (SED; Mognon et al., 2011) assesses the difference between the IC weights in the left and right fronto-temporal areas of the scalp. It was calculated according to Equation 4.

(4) $$SED=\;\left|\frac{1}{k}\sum_{e=1}^{k}a_{k,LE}-\frac{1}{k}\sum_{e=1}^{k}a_{k,RE}\right|$$

where *LE* is the left fronto-temporal area and includes the weights of electrodes whose angular positions range from −60 to −30° From nasion, *RE* is the right fronto-temporal area that includes the weights of electrodes whose angular positions range from 30 to 60°, and *a* is the vector of the IC weights in the *k* electrode positions within the *LE* and *RE* areas. In cases where the average weights of *LE* and *RE* were of the same sign, it was assumed no net horizontal eye movements were made and SED was set to zero. SED values were then normalized to the maximum SED value obtained across all ICs for a given dataset.

5–9. Power Spectral Densities (PSD):

To calculate the Power Spectral Density Features (PSD-Delta, PSD-Theta, PSD-Alpha, PSD-Beta, and PSD-Gamma) we first estimated the total PSD across the spectrum from 0.3 to 100 Hz for each IC time course (Welch, 1967). We then calculated the mean PSD in each of the following frequency bands: Delta band (0.3–4 Hz), Theta band (4–8 Hz), Alpha band (8–12 Hz), Beta band (12–40 Hz), and Gamma band (40–100 Hz). The PSD-Delta, PSD-Theta, PSD-Alpha, PSD-Beta, and PSD-Gamma features were then defined as the proportion of spectral power in their respective frequency bands of total spectral power across all bands.

10. Cardiac Identification Feature (CIF):

Although identification of artifacts generated from cardiac sources was not a specific aim of the present study, the Cardiac Identification Feature (CIF), an original feature of the Fingerprint Method designed to discriminate potential cardiac artifacts from other artifact types, was included. First, we defined a frequency band of 0.8–3.0 Hz that corresponds to a cardiac frequency range of 48–180 beats per minute (bpm). Although athletes can generally reach higher heart rates during intensive physical load, the volunteers participating in the cycling task experiment (during which EEG data were collected to validate the Optimized Fingerprint Method, see *cp*. 2.3.1) were asked to keep a pedaling rate of 80 rpm, which was not intensive physical load given their training level. Consequently, no increase of their heart rate was observed beyond 180 bpm. The maximum PSD peak in the frequency band was identified. Then, all the peaks in the IC time course corresponding to the maximum PSD peak were selected. If no maximum PSD peak was identified, *CIF* was set to 0. Among the peaks identified in the IC time course, those that were greater than half the average peak amplitude were retained, and only those that occurred at a distance corresponding to the expected inter-beat interval were considered. The cardiac identification feature (*CIF*) was then calculated according to Equation 5.

(5) $$\begin{array}{l} CIF=\frac{N_{fcp}}{N_{ecb}} \end{array}$$

where *N*_*fcp*_ is the number of the found cardiac peaks in the IC time course, and *N*_*ecb*_ is the number of expected cardiac beats based on the identified cardiac frequency (maximum PSD peak).

11. Myogenic Identification Feature (MIF):

Given that myogenic signals typically have frequency components >20 Hz (Goncharova et al., 2003; Whitham et al., 2007), for each IC we calculated the PSD in two frequency bands: 0–20 Hz and 21–100 Hz. If the PSD in the frequency band 0–20 Hz was greater than the PSD in the frequency band 21–100 Hz, the analyzed IC was considered to not contain a myogenic artifact, and *MIF* was set to 0. Otherwise, MIF was calculated according to Equation 6.

(6) $$MIF=\frac{\sum_{\text{21Hz}}^{\text{100Hz}}\text{PSD}}{\sum_{\text{0Hz}}^{\text{20Hz}}\text{PSD}+\sum_{\text{21Hz}}^{\text{100Hz}}\text{PSD}}$$

12. Eye Movement Correlation Feature (EM-CORR):

A characteristic left-to-right horizontal eye movement template was generated from multiple exemplary recordings of horizontal eye movement artifacts using 4 s time windows. This template was compared to the IC time course using a sliding window procedure: the template was compared to the IC time course using a 4 s time window which was advanced by one data point at a time until the entire IC time course was spanned. A vector of linear correlation values was obtained by calculating the Pearson product moment correlation coefficient between the template and each moving window of the IC. To account for horizontal eye movements in both directions, we calculated the absolute value of the correlation coefficients. Only the correlation coefficients with an absolute value ≥0.65 were retained to calculate the correlation feature, EM-CORR, which was estimated as the average of the retained absolute correlation values, as given by Equation 7.

(7) $$\begin{array}{l} CORR=\frac{\sum_{i=1}^{N}\left(r_{i}\right)}{N} \end{array}$$

where *r*_*i*_ is the absolute value of the retained correlation coefficient for the *i*th IC 5 s window, and *N* is the total number of retained correlation values. If no absolute correlation value was ≥0.65, then EM-CORR was set to 0.

13. Eyeblink Correlation Feature (EB-CORR):

The calculation of the Eyeblink Correlation Feature (EB-CORR) was identical to the calculation of EM-CORR except that each IC time course was compared to a template of eyeblinks generated from multiple eyeblink artifacts using 4 s time windows.

14. Entropy Feature (EF):

IC time courses were epoched into 5 s non-overlapping consecutive segments. The Entropy (H) of each IC was then calculated according to Equation 8.

(8) $$\begin{array}{l} H^{i}\left(j\right)=-\underset{x\in j}{\sum }p_{j}^{i}\left(x\right)log\left(p_{j}^{i}\left(x\right)\right) \end{array}$$

where *j* represents the *j*th 5 s segment of the *i*th IC, and $p_{j}^{i}\left(x\right)$ is the probability of observing the activity values *x* in the distribution of activity in the *j*th 5 s segment of the *i*th IC. After calculating these entropy measures for all segments and all ICs, we normalized the segment-entropy measures to 0 (mean) and 1 (standard deviation) for each segment across all ICs. For each IC, we then calculated the number of entropy measures ≥1.64 or ≤ −1.64. The Entropy Feature (EF) was then defined according to Equation 9.

(9) $$\begin{array}{l} EF=\;\frac{N_{sig}}{N_{tot}} \end{array}$$

where *N*_*sig*_ is the number of entropy measures ≥1.64 or ≤ −1.64, and *N*_*tot*_ is the total number of entropy measures for the given IC. Values of EF ≤ 0.2 were set to 0.

#### Optimization of the fingerprint method

Once the features of each IC from each artifact dataset had been calculated and all ICs were labeled, we used these data to create a set of automatic classifiers (one for each artifact type) which would be capable of classifying artifacts in new data collected during a sports science protocol (see *cp*. 2.3.1 and 2.3.2). For this purpose, we chose to use a set of nonlinear binary SVMs, each of which used a radial basis function kernel (Aizerman et al., 1964). Supervised SVM classifiers are binary classifiers that, given a set of training examples (ICs) defined by a set of parameters (the fingerprint features) preliminarily labeled as belonging to one or the other of two classes (the expert labels), are able to build a model which assigns new data to one of the two classes by mapping the training examples as points in an *n*-dimensional feature space, where *n* is the number of fingerprint features. The model determines a hyperplane (decision boundary) by maximizing the margin between points representing the two classes (Vapnik, 1995, 1998). The SVM classifier then determines the class of new data based on its position in the feature space relative to the decision boundary. We chose to use SVM classifiers to optimize our Fingerprint Method for several reasons: First, SVMs have proven effective in the automatic classification of EEG artifacts in a number of applications, including our own work (e.g., De Martino et al., 2007; Halder et al., 2007; Tamburro et al., 2018). Additionally, SVM classifiers can learn in sparse, high-dimensional spaces with few training examples (Yao et al., 2001). Given that our goal is to evaluate multiple feature dimensions using the available training data, these characteristics make SVMs particularly attractive. Finally, since the optimization procedure requires the training and testing of a large number of classifiers we preferred SVMs over other machine learning methods, such as artificial neural networks, because SVM classifiers are easily modified with relatively low computational load. All SVM models were generated using functions in the Matlab Statistics and Machine Learning Toolbox (v. R2016b; MathWorks, Inc., Natick, Massachusetts, United States).

For each SVM artifact classifier, we wanted to determine the set of fingerprint features which best classified each artifact type. Specifically, we implemented an optimization procedure where first we created a set of SVM classifiers which tested all possible combinations of parameters (exhaustive search) and then examined each classifier and selected the optimal classifier by applying a set of evaluation criteria (fitness evaluation). We anticipated that a unique set of parameters may be optimal for each of the three artifact types; therefore, the optimization procedure was performed independently for each artifact type using artifact type-specific datasets.

Our optimization procedure consisted of six steps and is outlined as follows:

1. Training and Testing Data Selection: For each artifact type, six datasets of the artifact EEG data were selected at random to test and evaluate the corresponding SVM classifier (testing set). The remaining artifact datasets were used to train the classifier (training set).
2. SVM Classifier Training: A set of SVM classifiers were built from the training set of data, where each classifier was trained using a different combination of fingerprint features. There are 16,383 unique possible combinations of the 14 IC-Fingerprint features, including those combinations containing only a single feature and the combination that includes all 14 features. Therefore, we generated a set of 16383 unique SVM classifiers. The expert labels were used to train each classifier to label each IC as “artifact” or “non-artifact.”
3. SVM Classifier Testing: Once each classifier had been trained, it was used to automatically classify artifactual and non-artifactual ICs in the testing data. Since a different set of features was used to train each classifier, the same set of features within the testing data was used in the automatic classification phase. Each IC of the testing datasets was automatically classified either as “artifact” or “non-artifact.”
4. SVM Classifier Assessment: The SVM classifications of each testing set IC were compared to the original labels provided by the experts. Four possible outcomes existed for each IC evaluated:
  1. In cases where both the classifier label and the expert label were “artifact,” the outcome was designated as true positive (TP);
  2. When both the classifier and the expert labels were “non-artifact,” the outcome was designated a true negative (TN);
  3. If the SVM classified the IC as “artifact” and the expert classified the IC as “non-artifact”, the outcome was designated a false positive (FP);
  4. If classifier classified the IC as “non-artifact” and the expert classified the IC as “artifact”, the outcome was designated a false negative (FN).

The accuracy of each classifier was assessed based on the probability that the classifier correctly labeled each IC in the testing set of data, which is given by the ratio of all correct outcomes to all observations according to Equation 10.

(10) $$\begin{array}{l} p\left(correct\;classification\right)=Accuracy\\ \;=\frac{\sum TP+\sum TN}{\sum TP+\sum TN+\sum FP+\sum FN} \end{array}$$

The preceding procedure (from 1 to 4) was performed ten separate times for each SVM classifier. On each iteration, a new random testing set of the six datasets was selected and each classifier was re-trained with the remaining training datasets. We chose to perform the cross-validation with ten iterations and selected six testing sets for each iteration to maintain consistency and allow comparisons with our previous findings (Tamburro et al., 2018).

5) SVM Classifier Optimization: Determining the optimal classifier was based on three criteria: (1) generalizability–performing well across different training and testing dataset combinations, (2) performance–achieving the highest accuracy, and (3) efficiency–achieving high accuracy with the fewest number of parameters. Therefore, finding the optimal classifier occurred in three stages: In stage one, we found all those classifiers which had achieved an accuracy equal to or exceeding the classifier that included all 14 fingerprint parameters (the full parameter classifier) in every iteration. If no classifiers were found at this stage, it was assumed that the full parameter classifier was the optimal classifier, and the optimization procedure was terminated. Otherwise, we continued to the second stage, where we found the classifiers from stage one that achieved the greatest summed accuracies across all ten iterations. In the third stage, we found the classifier from the second stage which used the fewest number of fingerprint parameters. This classifier was then chosen as the final (optimal) SVM classifier for that artifact type. In the event that more than one classifier met these criteria, additional iterations were performed, and the classifiers meeting criteria were again evaluated until only one met the optimization criteria.

The three final SVM classifiers—the eyeblink artifact SVM classifier, the eye movement artifact SVM classifier, and the myogenic artifact SVM classifier—which had been selected from the optimization procedure, were re-trained using all artifactual EEG datasets (i.e., the training and testing sets were re-combined). These three classifiers comprised the final model of the Optimized Fingerprint Method.

### Validation of the optimized fingerprint method in sports application data

#### Experimental design

**Figure 1:**
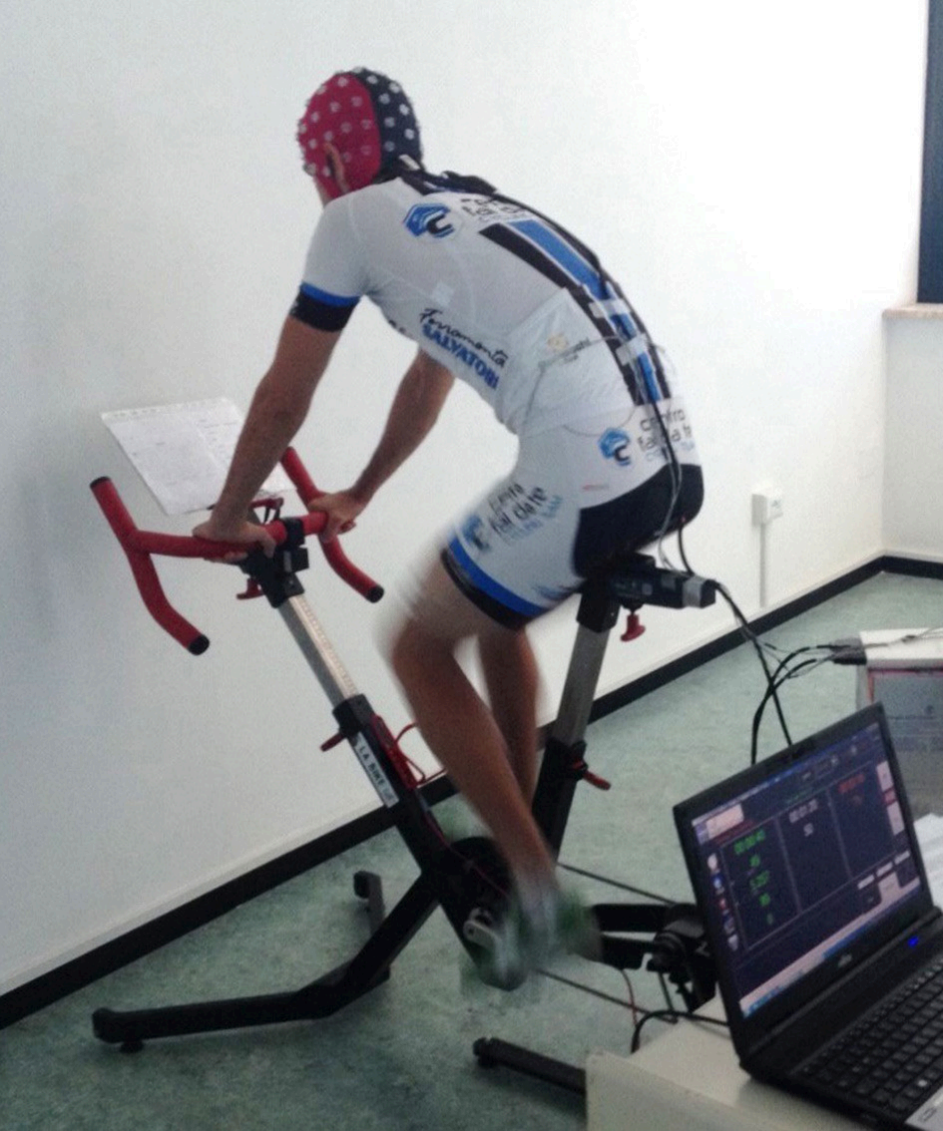
A participant performing the cycling endurance task during EEG data acquisition. Written informed consent was obtained from this participant for the publication of this image.

For evaluation of the Optimized Fingerprint Method in experimental EEG, we used data collected during a sports performance paradigm where participants performed an endurance cycling task. During the task, participants performed a time-to-exhaustion cycling task on a stationary cycling machine (MagneticDays Cyclo-ergometer, ORFSrl, Arezzo, Italy) while EEG, electrocardiographic (ECG) recordings, and bilateral electromyogenic (EMG) measures of the outer thigh (vastus lateralis) muscles were recorded. The task began with an initial subjective report of perceived physical exertion (Borg, 1982). Participants then began pedaling and were asked to maintain a cycling rate of at least 80 revolutions per minute (rpm) with the cycloergometer initially set to produce a 50 Watt load. Every 60 s, participants again reported their perceived physical exertion, and the cycloergometer load was increased by 25 Watts. Cycling continued until participants reported maximum perceived exertion and were unable to sustain a cycling rate of 80 rpm. Please note that, at a constant pedaling rate of 80 rpm, athletes' heart rates remained below 180 bpm (the range specified for the Cardiac Artifact Feature, see *cp*. 2.2.4). Figure 1 illustrates an example of a participant performing the cycling task.

#### EEG acquisition and pre-processing

EEG data were acquired in separate sessions using either a commercial gel-based electrode system or an experimental novel dry electrode system. The commercial system comprised 64 Ag/AgCl electrodes in an adapted 10–10 montage (Waveguard, Advanced Neuro Technologies B.V., Enschede, Netherlands). The dry electrode system comprised 64 multipin polyurethane electrodes with a Ag/AgCl coating (Fiedler et al., 2015) arranged in a quasi-equidistant montage (see Supplementary Materials). Please note that both systems differ from those used to acquire the cued artifactual data (see systems' layouts in the Figure S1). Electrolytic gel (ECI-Electrogel, Electrocap International Inc., USA) was applied at each electrode location for the standard system only. For all acquisitions, EEG signals were recorded using a unipolar biosignal amplifier at a sampling frequency of 1024 Hz (eego™ sports, Advanced Neuro Technologies B.V., Enschede, Netherlands). A standard Ag/AgCl electrode applied over the left mastoid served as ground electrode. A second standard Ag/AgCl electrode applied over the right mastoid served as reference electrode.

In total, 41 EEG datasets were acquired (20 using gel-based electrodes, 21 using dry electrodes). EEG data were bandpass filtered offline using cutoff frequencies at 0.3 and 100 Hz, and a notch filter at 50 Hz. EEG data were visually inspected, and isoelectric (saturated) channels and those exhibiting poor scalp-surface contact or excessive noise throughout the time course were excluded (McMenamin et al., 2010). Additionally, time segments exhibiting temporary electrode displacement or excessive noise due to non-physiological artifacts (e.g., electrode cable tension) in more than 30% of the channels were trimmed from the data.

#### IC parameterization and expert artifact classification

Datasets were decomposed into 20 or 50 ICs by performing ICA with PCA pre-whitening. For each dataset, the decomposition level depended on the number of retained channels (see *cp*. 2.3.2). The ICs from each dataset were then parameterized into 14 IC-Fingerprint features using a process identical to that described in section Identification of Fingerprint Features. Additionally, the time course, topological scalp map, and power spectrum of each IC were examined and each IC from each dataset was labeled as either “eyeblink artifact,” “eye movement artifact,” “myogenic artifact,” or “non-artifact.” Note that, unlike the EEG datasets containing cued artifacts of only one type, each of these EEG datasets could contain all three artifact types, consistent with what would be expected from real data acquired in an experimental setting.

#### Automatic artifact classification and evaluation

A subset of datasets was selected for testing the Optimized Fingerprint Method. Visual inspection of the EEG traces revealed that several datasets were affected by significant data loss in the time courses likely due to electrode displacement. Therefore, it was determined that the best 12 datasets would be retained for the automatic artifact classification. These datasets displayed <50% of channels affected by gross artifacts of non-physiological mechanical and hardware origin (which were removed before signal decomposition), and produced ICs with clearly identifiable artifacts in both the time courses and the IC topographies. All EEG datasets were selected prior to the evaluation of the Optimized Fingerprint Method.

Twelve EEG datasets (ten from conventional gel-based electrode system acquisitions and two from dry electrode system acquisitions) were retained for validation of the Optimized Fingerprint Method. There was a total of 360 ICs from all datasets including 13 eyeblink artifactual ICs, 15 eye movement artifactual ICs, and 106 myogenic artifactual ICs. The average duration of the experimental datasets was 235.33 ± 143.30 s (mean ± S.D).

The Optimized Fingerprint Method was then applied to the selected experimental datasets. Each of the three artifact classifiers was applied separately and independently to classify only one artifact type: The eyeblink artifact classifier was applied to the datasets to detect eyeblink artifactual ICs, the eye movement classifier was applied to detect eye movement artifactual ICs, and the myogenic classifier was applied to detect myogenic artifactual ICs. Therefore, each classifier detected only one type of artifact and labeled the other ICs as “non-artifact.”

The performance of each classifier was evaluated separately by comparing the automatic classifier labels to the labels assigned to each IC in the experimental datasets by the expert investigators and assessing the accuracy of the classifier according to Equation 10.

We included two additional measures of each classifier's performance in detecting artifacts in the experimental datasets. First, we ascertained the conditional probability that an IC is truly artifactual given that the SVM classifier labels it as an “artifact,” which is defined as the proportion of true positives to all outcomes where the classifier labeled the IC as an “artifact.” This probability is known as the Precision of the classifier and is defined by Equation 11.

(11) $$p(true\;artifact|“artifact”\;label)=Precision=\;\frac{\sum TP}{\sum TP+\sum FP}$$

Second, we calculated the conditional probability that an IC is truly artifactual given that the SVM classifier labeled it as “non-artifact,” which is known at the False Omission Rate, defined by Equation 12.

(12) $$\begin{array}{l} p(true\;artifact|“non-artifact”\;label)\\ =False\;Omission\;Rate=\;\frac{\sum FN}{\sum FN+\sum TN} \end{array}$$

In the current study, all truly artifactual ICs are known *a priori*. In real-world settings, where the true classification of an IC is presumed to be unknown, the Precision and False Omission Rates of the SVM classifiers give the probabilities that an IC is correctly classified based only on the labels given by the classifier.

## Results

### The optimized fingerprint method

#### Outcome of feature selection and evaluation of the optimized fingerprint method

The final sets of fingerprint features selected for inclusion in each SVM classifier are outlined in Table 2.

**Table 2:** Fingerprint features for artifact classifiers.

**Parameter**	**Eyeblink artifact classifier**	**Eye movement artifact classifier**	**Myogenic artifact classifier**
*K*	✓	✓	✓
*MEV*	✓	✓	✓
*SAD*	✓		✓
*SED*		✓	✓
*PSD DELTA*	✓	✓	✓
*PSD THETA*		✓	✓
*PSD ALPHA*			✓
*PSD BETA*		✓	✓
*PSD GAMMA*		✓	✓
*CIF*		✓	✓
*MIF*		✓	✓
*EM-CORR*		✓	✓
*EB-CORR*			✓
*EF*			✓

##### Final eyeblink artifact SVM classifier

Four features were selected for the final automatic eyeblink SVM classifier: Temporal Kurtosis, Maximum Epoch Variance, Spatial Average Distance, and the Delta Band PSD. The outcome of the eyeblink artifact feature selection procedure is summarized in Table 3. The accuracies obtained from the full feature classifier are included for comparison. The final classifier failed to correctly classify one eyeblink IC in iteration five and otherwise displayed perfect performance, producing a mean accuracy of 0.9997 (*SD* = 0.0009) across all iterations. The performance of the SVM classifier using the four selected features was significantly more accurate than that of the SVM classifier using the full feature classifier [*t*-test, paired samples; *t*_(9)_ = 2.34, *p* = 0.044].

**Table 3:** Outcome of the feature selection for the detection of eyeblink artifacts.

**Iteration number**	**No. ICs**	**No. artifacts**	**TP**	**TN**	**FP**	**FN**	**Accuracy (selected features)**	**Accuracy (all features)**
1	330	8	8	322	0	0	1.000	1.000
2	210	7	7	203	0	0	1.000	0.995
3	270	7	7	263	0	0	1.000	1.000
4	360	7	7	353	0	0	1.000	0.997
5	330	7	6	323	0	1	0.997	0.997
6	240	8	8	232	0	0	1.000	0.996
7	360	6	6	354	0	0	1.000	0.997
8	180	7	7	173	0	0	1.000	1.000
9	270	7	7	263	0	0	1.000	1.000
10	240	7	7	233	0	0	1.000	1.000
Mean	279	7.1					>0.999	0.998

##### Final eye movement artifact SVM classifier

Ten features were selected for the final eye movement artifact SVM classifier: Temporal Kurtosis; Maximum Epoch Variance; Spatial Eye Distance; PSD in the Delta, Theta, Beta, and Gamma Bands; The Cardiac and Myogenic Identification Features; and Eye Movement Correlation Feature. The outcome of the eye movement artifact feature selection procedure is summarized in Table 4 along with the accuracies from the full feature classifier. The mean accuracy of the final eye movement classifier across all iterations was 0.9380 (*SD* = 0.0170). As with the final eyeblink classifier, the final eye movement artifact classifier using the selected features was significantly more accurate than the full feature classifier [*t*-test, paired samples; *t*_(9)_ = 4.033, *p* = 0.003].

**Table 4:** Outcome of the feature selection for the detection of eye movement artifacts.

**Iteration number**	**No. ICs**	**No. artifacts**	**TP**	**TN**	**FP**	**FN**	**Accuracy (selected features)**	**Accuracy (all features)**
1	210	55	51	144	11	4	0.929	0.924
2	180	23	21	150	7	2	0.950	0.950
3	180	41	38	132	7	3	0.944	0.928
4	150	24	23	121	5	1	0.960	0.953
5	210	45	42	157	8	3	0.948	0.938
6	240	65	52	167	8	13	0.913	0.908
7	240	60	55	170	10	5	0.938	0.921
8	240	54	47	181	5	7	0.950	0.942
9	270	74	57	188	8	17	0.907	0.900
10	120	18	16	97	5	2	0.942	0.942
Mean	204	45.9					0.938	0.931

##### Final myogenic artifact SVM classifier

None of the classifiers which employed a subset of the fingerprint features achieved greater accuracies than the classifier employing the full set of features on every iteration, so the full set of features was retained in the final myogenic artifact SVM classifier. The mean accuracy of the final myogenic classifier across all iterations was 0.9622 (*SD* = 0.0153). The performance of the final myogenic artifact classifier is summarized in Table 5.

**Table 5:** Outcome of the feature selection for the detection of myogenic artifacts.

**Iteration number**	**No. ICs**	**No. artifacts**	**TP**	**TN**	**FP**	**FN**	**Accuracy**
1	270	83	79	184	3	4	0.974
2	150	51	50	95	4	1	0.967
3	240	68	63	171	1	5	0.975
4	240	93	89	144	3	4	0.971
5	240	78	76	147	15	2	0.929
6	210	46	45	156	8	1	0.957
7	240	81	80	154	5	1	0.975
8	180	76	72	98	6	4	0.944
9	270	93	89	170	7	4	0.959
10	240	82	79	154	4	3	0.971
Mean	228	75.1					0.962

### Validation of the optimized fingerprint method in sports application data

Table 6 summarizes the results of the validation of the Optimized Fingerprint Method in cyclist data. The eyeblink artifact SVM classifier correctly identified all non-artifactual ICs and 11 out of 13 eyeblink artifactual ICs giving the classifier an accuracy of 99.4%. The eyeblink classifier performed with perfect precision (Precision = 100.0%) and a False Omission Rate of 0.6%.

**Table 6:** Results of the validation of the optimized fingerprint method in cycling data.

	**Eyeblink artifact classification**	**Eye movement artifact classification**	**Myogenic artifact classification**
TP	11	11	90
TN	347	344	244
FP	0	1	10
FN	2	4	16
Accuracy	0.994	0.986	0.928
Precision	1.000	0.917	0.900
False omission rate	0.006	0.012	0.062

The eye movement classifier correctly identified 344 out of 345 non-artifactual ICs and 11 out of 15 eye movement artifactual ICs for an accuracy of 98.6%, a Precision of 91.7%, and a False Omission Rate of 1.2%.

The myogenic classifier correctly classified 244 out of 254 non-artifactual ICs and correctly identified 90 out of 106 myogenic artifactual ICs. The accuracy of the myogenic classifier was 92.8% with a Precision of 90.0% and a False Omission Rate of 6.2%.

**Figure 2:**
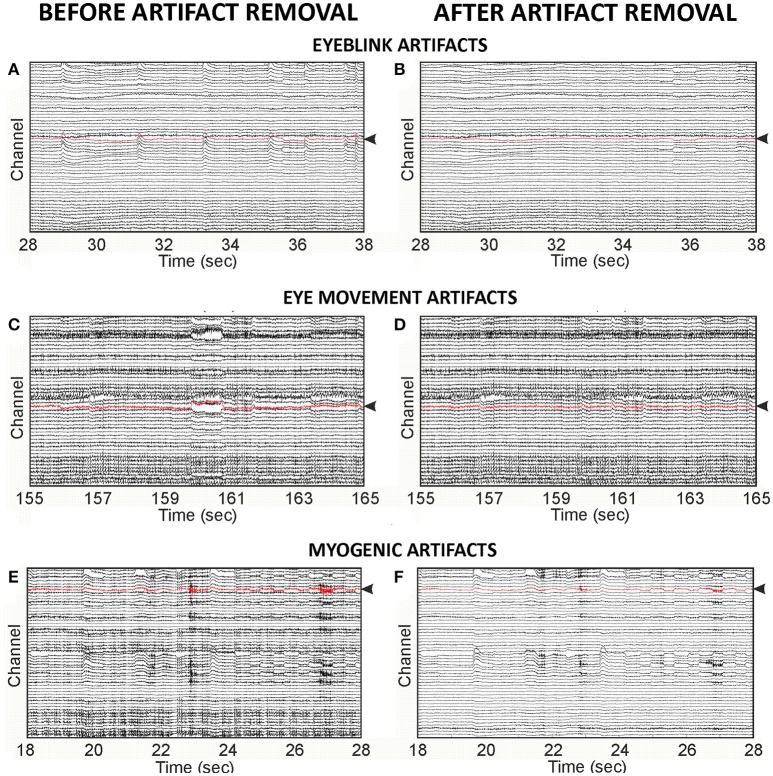
Examples of original EEG cyclist data and EEG data reconstructed after SVM classified artifactual ICs were removed. **(A)** EEG trace with eyeblink artifacts**:** Original data segment containing seven eyeblink artifacts. Average SNR = 14.23 dB. **(B)** EEG trace after eyeblink artifact removal**:** The same data segment shown in **(A)** reconstructed after removal of the ICs automatically classified as artifactual for eyeblinks. Average SNR = 4.64 dB. **(C)** EEG trace with eye movement artifacts**:** Original data segment containing one eye movement artifact (at~160 s). SNR = 5.92 dB. **(D)** EEG trace after eye movement artifact removal: The same segment shown in **(C)** reconstructed after removal of the ICs automatically classified as artifactual for eye movements. SNR = 1.18 dB. **(E)** EEG trace with myogenic artifacts: Original data containing two myogenic artifacts (at ~23 s and 27 s). Average SNR = 15.45 dB. **(F)** EEG trace after myogenic artifact removal: The same segment shown in **(E)** reconstructed after removal of the ICs automatically classified as artifactual for myogenic artifacts. Average SNR = 6.28 dB. In each EEG data segment, distance between electrodes (between each point on the abscissa) = 100 microvolts. Average SNR was calculated in the EEG channel highlighted in red shown in each EEG trace.

To assess the outcome of the Optimal Fingerprint Method on data quality, we compared the original cyclist EEG data to data reconstructed after the removal of the automatically classified artifactual ICs. Figure 2 illustrates examples of EEG traces before and after classified artifact removal. The improvement of EEG signal quality after artifact removal was estimated across a 10 s EEG data segment for each artifact type by the change in signal-to-noise ratio (SNR) between the original and reconstructed EEG data. In each EEG data segment, SNR was evaluated at a single channel where “signal” refers to the artifact-of-interest, and “noise” refers to baseline activity. The spontaneous EEG baseline activity (noise term of the SNR calculation) is intended as an estimate of brain activity present in the original and reconstructed signals. For each artifact in the EEG time segment, the absolute maximum voltage of a 200 ms baseline preceding the artifact was the noise value which was compared to the maximum voltage of the 200 ms time window centered on the adjacent artifact defining the signal amplitude. SNR was then calculated according to Tamburro et al. (2018) (Equation 15) and averaged across all artifacts in the EEG time segment.

## Discussion

The aim of the present study was to develop an automatic artifact classification method capable of accurately identifying eyeblink, eye movement, and myogenic artifacts in EEG data and to test the model with experimental EEG data acquired during a cycling endurance task. Our results indicate that the Optimized Fingerprint Method was highly successful in detecting artifacts in artifact-rich EEG testing data when an optimal set of identifying features was selected. This method also performed well in detecting physiological artifacts in experimental sports science EEG data, obtaining an accuracy of 92.8% when identifying muscle artifacts, and accuracies >98% when identifying eyeblink and eye movement artifacts.

### The optimal fingerprint features

One of the goals in optimizing the Fingerprint Method was to determine which features were best suited to identify different types of artifacts. We found that a unique cluster of spatial, temporal, spectral, and statistical features was optimal for selecting eyeblink and eye movement artifacts, whereas the full set of features had the best performance for detecting myogenic artifacts.

Two temporal features, Kurtosis and Maximum Epoch Variance, were useful in determining all artifact types under investigation. These features are sensitive to transient changes in the amplitudes of IC time courses where high values indicate rapid fluctuations in potential as occur during eyeblinks, eye movements, and transient muscle contractions. Low values indicate lower amplitude and continuous activity. Therefore, their selection in detecting artifacts is not surprising. However, it is important to note that these features were selected from a process that employed evoked artifact training and testing data where participants were cued to generate artifacts at intervals which were consistent with the epoch intervals used to calculate these features. Their efficacy in separating artifacts from cerebral activity during evoked tasks, such as in experiments using event-related designs where stimuli generate sensory or cognitive potentials at regular intervals, remains to be investigated.

All the final artifact SVM classifiers included in the Optimized Fingerprint Method also employed spatial and spectral features. Two spatial features, the Spatial Average Difference and the Spatial Eye Difference, are particularly sensitive to the detection of eyeblinks and eye movements, respectively, and their inclusion in the final eyeblink and eye movement artifact classifiers is not unexpected. The eyeblink artifact classifier also included a spectral power feature in the 0–4 Hz (Delta) range, which is the frequency band where eyeblink artifacts typically peak. The eye movement classifier included spectral density features in the Delta, Theta, Beta, and Gamma ranges. Interestingly, a spectral feature which we developed to specifically identify myogenic artifacts, the Myogenic Identification Feature, was also included in the set of features employed by eye movement classifier. The MIF calculates the ratio of high frequency (20–100 Hz) to low frequency (0–20 Hz) power, based on the observation that muscle interference in brain activity recordings tends to generate frequency components >20 Hz. The inclusion of the MIF and spectral power features from a range of frequencies suggests that, at least in the case of eye movement artifacts, both fine-grained and course spectral information prove useful in artifact detection and discrimination.

An unexpected outcome of our feature selection procedure was the inclusion of the Cardiac Identification Feature in both the eye movement and myogenic artifact classifiers. We developed this feature to specifically identify cardiac artifacts, and its usefulness in detecting eye movements and myogenic activity is unclear. The measure is based upon spectral power peaks in the lower Delta frequency band (0.8–3.0 Hz) which are not typically present in the generation of eye movement and muscle artifacts. However, it is worth noting that the inclusion of the CIF in the eye movement and myogenic artifact classifiers is consistent with the inclusion of the spectral power feature in the Delta range. Therefore, it may be possible that the CIF is useful in detecting cardiac artifacts as it was designed to do, and so the eye movement and myogenic classifiers may use information present in the CIF feature to differentiate cardiac artifacts from eye movements and muscle activity. We are currently developing a cardiac artifact classifier that is an improvement with respect to its former version (Tamburro et al., 2018). The performance of this upgraded classifier may elucidate the benefit of the Cardiac Identification Feature in the separation of multiple artifact types, especially when applied to the detection of cardiac artifacts in sports science settings.

In the final model, the full set of features were retained for classification of muscle artifacts by the myogenic artifact SVM classifier. Although other classifiers which were tested during the optimization procedure and which utilized fewer features for artifact detection achieved a higher average accuracy and outperformed the full feature classifier on many iterations of the feature selection procedure, our strict optimization criteria required equal or superior performance in every iteration. This criterion was imposed to ensure that our optimized classification model generalized well and was able to detect artifacts in a variety of different settings. There are several reasons why employing the full set of features was advantageous in detecting myogenic artifacts in our artifactual data. First, we trained the myogenic classifier using a variety of different muscle artifact types typically encountered in EEG data including jaw and facial muscle contractions, and head and neck movements. Each of these muscle artifact types possesses a unique set of spatial, temporal, spectral and statistical features. Therefore, while some sets of features may be optimal in detecting a specific type of muscle artifact, the full range of features may be required to detect all the myogenic artifacts encountered. Second, some of the features retained were designed to detect a broad range of artifactual and non-artifactual differences while other features were specifically designed to detect only one artifact type. Since the purpose of the myogenic classifier is not only to detect muscle artifacts but to correctly discriminate between muscle and non-muscle artifacts, including artifacts of other types, the full set of features may have been necessary to accurately identify differences between muscle artifacts and artifacts of other physiological origin. For example, muscle artifacts tend to be spatially focal and often appear in frontal scalp regions overlapping the channels used to calculate the Spatial Average Distance and the Spatial Eye Difference features. These features are based on specific differences in activity between frontal and posterior scalp regions and the values generated from focal muscle activity are likely different from those generated during ocular activity. Additionally, posterior muscle artifacts generated during neck movements will have null SAD and SED values. Therefore, the myogenic artifact classifier may exploit the information in these features to differentiate artifacts of myogenic origin from eyeblink and eye movement artifacts.

The Fingerprint Method combines features from other artifact detection methods with original features designed to maximally discriminate artifactual and non-artifactual ICs. While the rationale for including each of these features is based upon the well-known spectral, spatial, temporal, and statistical characteristics of artifactual data, to our knowledge, the Optimized Fingerprint Method is the first method to critically evaluate multiple features and select the best combination of those features to detect specific artifacts based on actual performance. This knowledge advances the field because it facilitates the development of more targeted features for artifact classification and aids researchers in identifying those characteristics of physiological artifacts which are most relevant for their detection and removal.

It should be noted that the Optimized Fingerprint Method is so far limited to the detection of only the most common types of physiological artifacts: eyeblinks, horizontal eye movements, and facial and neck muscle artifacts. The presence of additional artifacts produced from cardiac and respiratory interference, vertical and convergent eye movements, and other physiological and non-physiological sources have not been addressed in the current study. It is an open question whether the Optimized Fingerprint Method would have similar performance in detecting these other artifact types. Nevertheless, we believe that the feature optimization procedure employed here can readily be used in the development of automatic detection methods for a broad range of artifact types.

### Automatic EEG artifact detection in the cycling endurance task

The Optimized Fingerprint Method was tested in a set of experimental data acquired during a sports performance task (a cycling endurance paradigm). The results indicate that the method performed very well in correctly identifying physiological eyeblink and eye movement artifacts as well as multiple myogenic artifacts in these data, suggesting that the Optimized Fingerprint Method is a useful tool for the detection of these physiological artifacts in EEG data acquired during sports applications.

The performance of the eyeblink SVM classifier when applied to the data acquired during a sports performance task (experimental data) was equivalent to the overall performance of the classifier in the EEG datasets with cued artifacts (artifact EEG datasets), while the eye movement classifier performed better in detecting eye movements in the experimental data than the average performance in cued artifact EEG datasets. The performance of the myogenic artifact classifier was slightly lower than its performance in the cued artifact EEG datasets; nevertheless, the classifier was able to correctly identify over 90% of the muscle artifact components present in these data. This is an encouraging result, given the considerable number and variety of muscle artifact components generated during cycling performance. The cycling task, which requires participants to cycle continuously with increasing effort, elicited sustained muscle tension in many cases, particularly in neck muscles used to maintain head posture and tone during continuous cycling. The myogenic artifact classifier, which was trained using data acquired during periodic muscle tension and relaxation intervals, might not be ideal in such cases. However, it was able to detect artifacts arising from sustained muscle tension.

EEG acquisition during sports performance tasks typically generates a variety of physiological artifacts which are not well managed by standard artifact minimization techniques due to task demands and data quality control issues. Indeed, the ubiquity of artifacts has historically been prohibitive to the design, collection, and analysis of EEG data in sports settings (Thompson et al., 2008). To date, there have only been a few attempts to automatically classify artifacts in EEG sports applications. In their review, Reis et al. (2014) recommend using the MARA automatic artifact removal software developed by Winkler et al. (2011) to detect and remove artifacts in sport related studies. However, to our knowledge, this software has not been specifically tested in EEG sports applications. In 2013, Gabsteiger and colleagues used an SVM classifier to automatically classify myogenic artifactual ICs in a study with specific neck and body movement exercises (Gabsteiger et al., 2014). These authors obtained good results when they evaluated their classifier in a test dataset from the same study (achieving 93% sensitivity and 96% specificity). Compared to our results, their classifier achieved higher sensitivity than ours [we achieved 85% sensitivity measured as (TP/(TP+FN))] and equivalent specificity [96% measured as (TN/(TN+FP))]. However, their results were limited to one subject's dataset taken from the same study, and the authors admit that unfamiliar exercises could result in worse performance. Further, these authors did not evaluate the identification and removal of other physiological artifacts, such as eyeblinks and eye movements. However, it should be noted that the scope of the present work was limited to addressing only the performance of our method. It remains an outstanding question how the Optimized Fingerprint Method directly compares to the method of Gabsteiger et al or other methods of automatic artifact removal in sports science since no direct comparisons between methods on the same data were performed. Indeed, it may be possible that other methods are superior in removing particular artifact types under certain conditions, or that a combination of methods yields the best results. We anticipate that, as the application of EEG in sports science becomes more commonplace, a broader range of artifact removal techniques will be applied and evaluated. Nevertheless, we think that the results we obtained in testing the Optimized Fingerprint Method in sports performance data represent a significant step forward in the search for effective methods to address physiological artifact contamination in EEG sports science applications.

The ultimate goal of any automatic EEG artifact classification system is to correctly identify and remove artifacts in EEG datasets with minimal human intervention. Ideally, such models would facilitate EEG data processing without appealing to human experience and expertise in identifying artifacts or depending on other modes of artifact reduction, such as minimizing artifact generation during data acquisition or monitoring artifacts via concurrent ocular, cardiac and myogenic recordings. In this scenario, investigators who depend on the model alone to correctly identify artifacts, would benefit from some measure of the reliability of the model's outcome. Therefore, we included measures of the precision and false omission rates in evaluating the performance of our model in experimental data. These measures inform potential users of the likelihood that a data component is actually artifactual depending on the label the model assigns to the component. Results indicate that our model achieved high precision (≥90%) and low false omission rates (0.6% in eyeblink misclassification, 1.2% in eye movement misclassification, 6.2% in myogenic misclassification) suggesting that our Optimized Fingerprint Method would be suitable as an automatic artifact classification tool in sports science research settings. On-going research seeks to achieve comparable measures in clinical research settings, thereby expanding the utility of the model across a variety of EEG research applications. Moreover, in other applications where only eyeblink or eye movement artifacts are a concern, the Optimized Fingerprint Method will prove more computationally efficient because fewer features will need to be calculated which may reduce computational cost by as much as 44%. Overall, the successful application of the Optimized Fingerprint Method to real-world experimental data is a proof-of-principle that the method generalizes well in a variety of domains.

## Ethics statement

This study was carried out in accordance with the recommendations of the Guidelines for Sperimentazione clinica non farmacologica no profit, monocentrica, University G. d'Annunzio of Chieti-Pescara (Italy) with written informed consent from all subjects. All subjects gave written informed consent in accordance with the Declaration of Helsinki. The protocol was approved by the University G. d'Annunzio of Chieti-Pescara (Italy) with Ethical Application Ref. n. 10-21/05/2015.

## Author contributions

All co-authors contributed to the study design. DS designed the procedure for the Optimized Fingerprint Method, developed the software, analyzed the data and wrote the manuscript. GT contributed to the development of the software. GT and PF collected all EEG datasets. SC supervised all phases of the work. All co-authors evaluated the results and revised the manuscript.

### Conflict of interest statement

The authors declare that the research was conducted in the absence of any commercial or financial relationships that could be construed as a potential conflict of interest.

## Supplementary material

Figure S1 Dry and gel-based (“wet”) electrode montages worn by participants during training data acquisitions **(a,b)** and during cycling data acquisitions **(c,d)**.
