# Supplementary figures and images for: Automatic Removal of Physiological Artifacts in EEG: The Optimized Fingerprint Method for Sports Science Applications
Source: Front Hum Neurosci. 2018 Mar 21;12:96. doi: 10.3389/fnhum.2018.00096 (PMC5871683; doi:10.3389/fnhum.2018.00096)

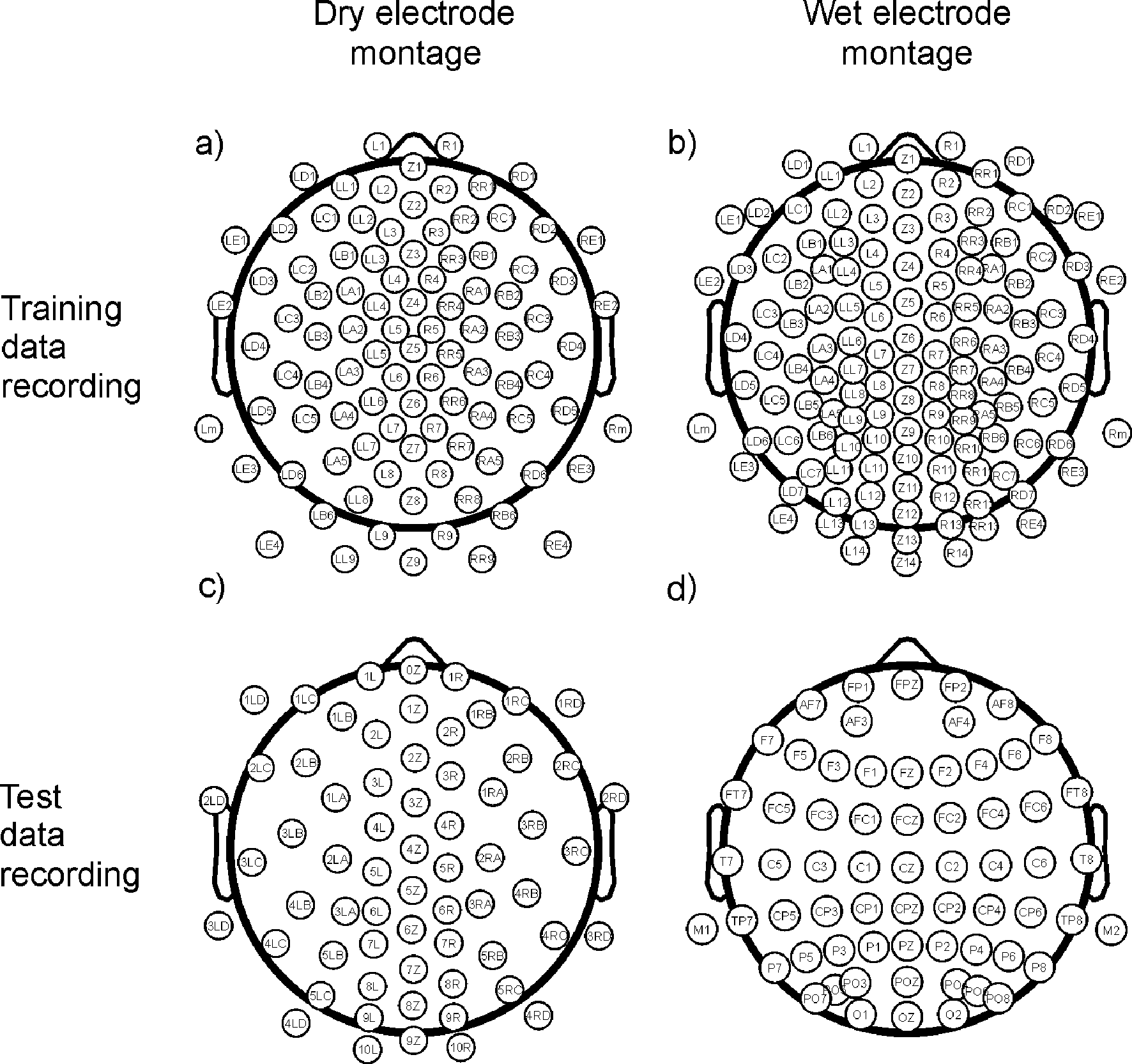

Supplement: Figure S1 — Dry and gel-based (“wet”) electrode montages worn by participants during training data acquisitions (a,b) and during cycling data acquisitions (c,d). [file Image1.TIF]
